# Supplementary figures and images for: Healthcare resource use and associated costs in a cohort of hospitalized COVID-19 patients in Spain: A retrospective analysis from the first to the third pandemic wave. EPICOV study
Source: PLoS One. 2023 Jan 25;18(1):e0280940. doi: 10.1371/journal.pone.0280940 (PMC9876243; doi:10.1371/journal.pone.0280940)

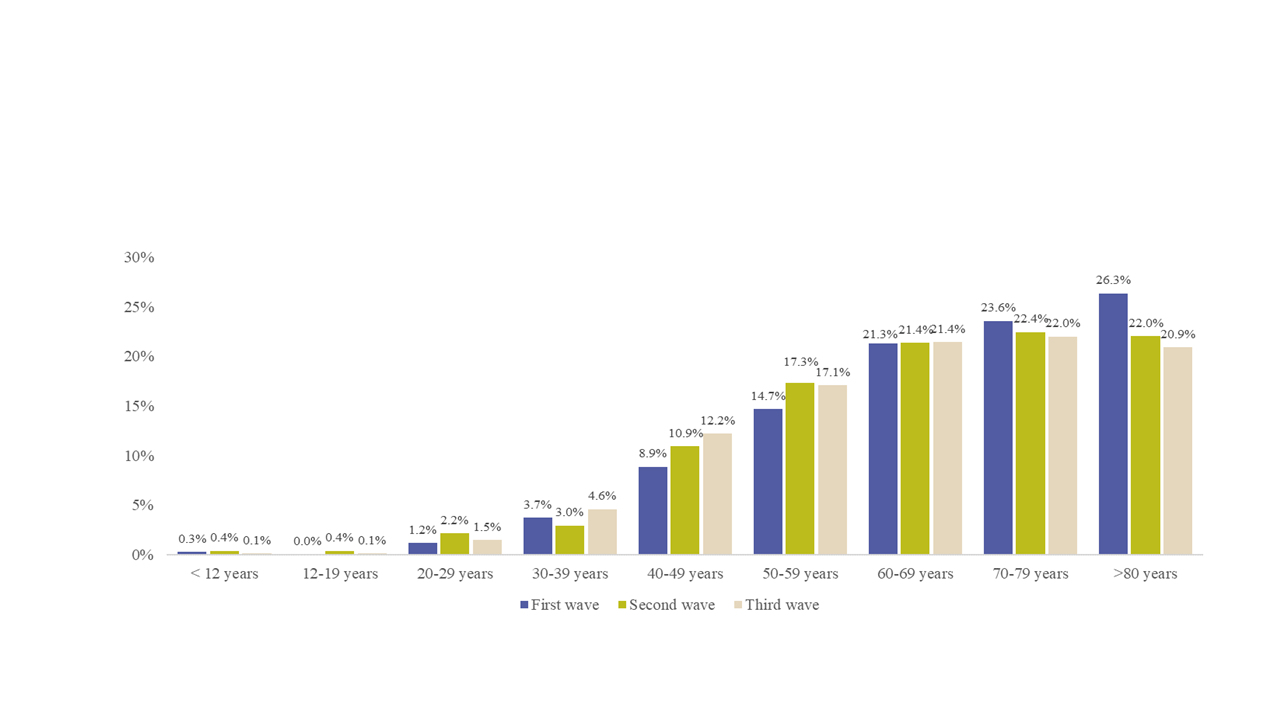

Supplement: S1 Fig — (TIF) [file pone.0280940.s001.tif]
