# Supplementary material for: Healthcare resource use and associated costs in a cohort of hospitalized COVID-19 patients in Spain: A retrospective analysis from the first to the third pandemic wave. EPICOV study
Source: PLoS One. 2023 Jan 25;18(1):e0280940. doi: 10.1371/journal.pone.0280940 (PMC9876243; doi:10.1371/journal.pone.0280940)
Supplement: S1 Table — (DOC) [file pone.0280940.s002.doc]

**S1 Table.** STROBE Checklist.

| **Section/item** | **Item No** | **Recommendation** | **Reported on page N/line N** |
| --- | --- | --- | --- |
| **Title/abstract** | 1a | Indicate the study’s design with a commonly used term in the title or the abstract | Page 1 (title)/Page 2/(abstract) |
|  | 1b | Provide in the abstract an informative and balanced summary of what was done and what was found | Page 2 |
| **Introduction** |  |  |  |
| Background/rationale | 2 | Explain the scientific background and rationale for the investigation being reported. | Page 3-4 |
| Objectives | 3 | State specific objectives, including any prespecified hypotheses | Page 4 |
| **Methods** |  |  |  |
| Study design | 4 | Present key elements of study design early in the paper | Page 4 |
| Setting | 5 | Describe the setting, locations, and relevant dates, including periods of recruitment, exposure, follow-up, and data collection. | Page 5 (study periods) |
| Participants | 6a | Cohort study—Give the eligibility criteria, and the sources and methods of selection of participants. Describe methods of follow-up | Page 5 (eligibility) |
|  | 6b | Cohort study—For matched studies, give matching criteria and number of exposed and unexposed | Does not apply |
| Variables | 7 | Clearly define all outcomes, exposures, predictors, potential confounders, and effect modifiers. Give diagnostic criteria, if applicable. | Page 7 (variables) |
| Data sources/ measurement | 8* | For each variable of interest, give sources of data and details of methods of assessment (measurement). Describe comparability of assessment methods if there is more than one group. | Page 7 (data source)  Comparability does not apply (descriptive) |
| Bias | 9 | Describe any efforts to address potential sources of bias. | Does not apply (descriptive) |
| Study size | 10 | Explain how the study size was arrived at | Does not apply (descriptive) |
| Quantitative variables | 11 | Explain how quantitative variables were handled in the analyses. If applicable, describe which groupings were chosen and why. | Page 8 (quantitative variables)  Group-ing does not apply (descriptive) |
| Statistical methods | 12a | Describe all statistical methods, including those used to control for confounding | Pages 8-10 (descriptive analysis) |
|  | 12b | Describe any methods used to examine subgroups and interactions | Does not apply (descriptive) |
|  | 12c | Explain how missing data were addressed | Does not apply |
|  | 12d | Cohort study—If applicable, explain how loss to follow-up was addressed | Does not apply |
|  | 12e | Describe any sensitivity analyses | Page 10 |
| **Results** |  |  |  |
| Participants | 13a* | Report numbers of individuals at each stage of study—eg numbers potentially eligible, examined for eligibility, confirmed eligible, included in the study, completing follow-up, and analysed | Page 5 and Figure 1 |
|  | 13b* | Give reasons for non-participation at each stage | Figure 1 |
|  | 13c* | Consider use of a flow diagram | Figure 1 |
| Descriptive data | 14a* | Give characteristics of study participants (eg demographic, clinical, social) and information on exposures and potential confounders | Pages 11-13 and Table 1 |
|  | 14b* | Indicate number of participants with missing data for each variable of interest | Does not apply |
|  | 14c* | Cohort study—Summarise follow-up time (eg, average and total amount) | Does not apply |
| Outcome data | 15* | Cohort study—Report numbers of outcome events or summary measures over time | Does not apply |
| Main results | 16a* | Give unadjusted estimates and, if applicable, confounder-adjusted estimates and their precision (eg, 95% confidence interval). Make clear which confounders were adjusted for and why they were included | Does not apply |
|  | 16b* | Report category boundaries when continuous variables were categorized | Results section (Pages 11-19) |
|  | 16c* | If relevant, consider translating estimates of relative risk into absolute risk for a meaningful time period | Does not apply |
| Other analyses | 17 | Report other analyses done—eg analyses of subgroups and interactions, and sensitivity analyses | Page 19 |
| **Discussion** |  |  |  |
| Key results | 18 | Summarise key results with reference to study objectives | Discussion section (Pages 20-26) |
| Limitations | 19 | Discuss limitations of the study, taking into account sources of potential bias or imprecision. Discuss both direction and magnitude of any potential bias | Pages 25-26 |
| Interpretation | 20 | Give a cautious overall interpretation of results considering objectives, limitations, multiplicity of analyses, results of similar studies, and other relevant evidence | Discussion section (Pages 20-26) |
| Generalisability | 21 | Discuss the generalisability (external validity) of the study results | Page 24 (limitation) |
| **Other information** |  |  |  |
| Funding | 22 | Give the source of funding and the role of the funders for the present study and, if applicable, for the original study on which the present article is based | Financial Disclosure section of the submission form |

*Give information separately for cases and controls in case-control studies and, if applicable, for exposed and unexposed groups in cohort and cross-sectional studies.
