# Supplementary material for: Healthcare resource use and associated costs in a cohort of hospitalized COVID-19 patients in Spain: A retrospective analysis from the first to the third pandemic wave. EPICOV study
Source: PLoS One. 2023 Jan 25;18(1):e0280940. doi: 10.1371/journal.pone.0280940 (PMC9876243; doi:10.1371/journal.pone.0280940)
Supplement: S2 Table — (DOC) [file pone.0280940.s003.doc]

**S2 Table**. Distribution of number of secondary diagnoses according to vaccination age bands

| **Secondary diagnoses** | < 12 years | 12-19 years | 20-29 years | 30-39 years | 40-49 years | 50-59 years | 60-69 years | 70-79 years | > 80 years | Total |
| --- | --- | --- | --- | --- | --- | --- | --- | --- | --- | --- |
| Certain infectious and parasitic diseases (A00-B99) | 6 | 0 | 24 | 71 | 174 | 289 | 424 | 507 | 492 | 1987 |
| Neoplasms (C00-D48) | 0 | 1 | 0 | 1 | 14 | 50 | 109 | 194 | 132 | 501 |
| Diseases of the blood and blood-forming organs and certain disorders involving the immune mechanism (D50-D89) | 2 | 1 | 4 | 9 | 38 | 60 | 90 | 158 | 207 | 569 |
| Endocrine, nutritional and metabolic diseases (E00-E89) | 0 | 0 | 8 | 35 | 132 | 299 | 613 | 879 | 904 | 2870 |
| Mental, Behavioral and Neurodevelopmental disorders (F01-F99) | 0 | 1 | 10 | 18 | 38 | 74 | 130 | 184 | 296 | 751 |
| Diseases of the nervous system (G00-G99) | 1 | 0 | 1 | 5 | 23 | 61 | 119 | 190 | 278 | 678 |
| Diseases of the eye and adnexa (H00-H59) | 2 | 0 | 0 | 0 | 3 | 10 | 11 | 29 | 101 | 156 |
| Diseases of the ear and mastoid process (H60-H95) | 0 | 0 | 1 | 2 | 2 | 5 | 11 | 27 | 83 | 131 |
| Diseases of the circulatory system (I00-I99) | 0 | 0 | 4 | 6 | 81 | 210 | 527 | 921 | 1518 | 3267 |
| Diseases of the respiratory system (J00-J99) | 5 | 3 | 22 | 83 | 311 | 544 | 772 | 923 | 1071 | 3734 |
| Diseases of the digestive system (K00-K94) | 0 | 0 | 2 | 12 | 53 | 128 | 201 | 274 | 371 | 1041 |
| Diseases of the skin and subcutaneous tissue (L00-L99) | 1 | 1 | 3 | 4 | 6 | 18 | 31 | 45 | 68 | 177 |
| Diseases of the musculoskeletal system and connective tissue (M00-M99) | 0 | 0 | 1 | 6 | 26 | 72 | 130 | 172 | 330 | 737 |
| Diseases of the genitourinary system (N00-N99) | 2 | 0 | 1 | 2 | 12 | 50 | 146 | 240 | 410 | 863 |
| Pregnancy, childbirth and the puerperium (O00-O9A) | 0 | 0 | 14 | 44 | 9 | 2 | 0 | 0 | 0 | 69 |
| Certain conditions originating in the perinatal period (P00-P96) | 1 | 0 | 0 | 1 | 0 | 0 | 0 | 0 | 0 | 2 |
| Congenital malformations, deformations and chromosomal abnormalities (Q00-Q99) | 0 | 0 | 0 | 0 | 0 | 0 | 0 | 0 | 0 | 0 |
| Symptoms, signs and abnormal clinical and laboratory findings, not elsewhere classified (R00-R99) | 2 | 2 | 10 | 43 | 98 | 194 | 255 | 280 | 400 | 1284 |
| Injury, poisoning and certain other consequences of external causes (S00-T88) | 0 | 1 | 0 | 5 | 5 | 18 | 30 | 35 | 71 | 165 |
| External causes of morbidity (V00-Y99) | 0 | 0 | 0 | 0 | 2 | 10 | 16 | 15 | 42 | 85 |
| Factors influencing health status and contact with health services (Z00-Z99) | 1 | 0 | 3 | 8 | 22 | 35 | 79 | 128 | 244 | 520 |
| TOTAL | **23** | **10** | **108** | **355** | **1049** | **2129** | **3694** | **5201** | **7018** | **19587** |
