# Supplementary material for: Healthcare resource use and associated costs in a cohort of hospitalized COVID-19 patients in Spain: A retrospective analysis from the first to the third pandemic wave. EPICOV study
Source: PLoS One. 2023 Jan 25;18(1):e0280940. doi: 10.1371/journal.pone.0280940 (PMC9876243; doi:10.1371/journal.pone.0280940)
Supplement: S3 Table — All patients. (DOC) [file pone.0280940.s004.doc]

**S3 Table**. Procedures during ED stay in the different outbreak waves. All patients.

| **Procedures at ED (ICD10*)** | **Procedures, N (%)** | | |
| --- | --- | --- | --- |
|  | **1^st^ Wave**  N = 2296 | **2^nd^ Wave**  N = 577 | **3^rd^ Wave**  N = 208 |
| Plain Radiography of Chest (BW03ZZZ) | 1374 (59.8) | 364 (63.1) | 136 (65.4) |
| Inspection of Tracheobronchial Tree, External Approach (0BJ0XZZ) | 428 (18.6) | 57 (9.9) | 21 (10.1) |
| Computerized Tomography of Chest and Abdomen (BW24ZZZ) | 182 (7.9) | 37 (6.4) | 17 (8.2) |
| Measurement of Arterial Saturation, Peripheral, Percutaneous Approach (4A033R1) | 77 (3.4) | 30 (5.2) | 5 (2.4) |
| Other procedures | 235 (10.2) | 89 (15.4) | 29 (13.9) |

Abbreviations: ICD10 (the 10th revision of the International Statistical Classification of Diseases and Related Health Problems); *Procedures were registered with the ICD-codes
