# Supplementary material for: Healthcare resource use and associated costs in a cohort of hospitalized COVID-19 patients in Spain: A retrospective analysis from the first to the third pandemic wave. EPICOV study
Source: PLoS One. 2023 Jan 25;18(1):e0280940. doi: 10.1371/journal.pone.0280940 (PMC9876243; doi:10.1371/journal.pone.0280940)
Supplement: S4 Table — All patients. (DOC) [file pone.0280940.s005.doc]

**S4 Table**. Procedures during hospital stay in the different outbreak waves. All patients.

| **Procedures during hospital stay (ICD10*)** | **Procedures, N (%)** | | |
| --- | --- | --- | --- |
|  | **1^st^ Wave**  N = 17 435 | **2^nd^ Wave**  N = 6718 | **3^rd^ Wave**  N = 6520 |
| Isolation (8E0ZXY6) | 2052 (11.8) | 638 (9.5) | 601 (9.2) |
| Introduction of Other Anti-infective into Peripheral Vein, Percutaneous Approach (3E03329) | 1722 (9.9) | 622 (9.3) | 626 (9.6) |
| Plain Radiography of Chest (BW03ZZZ) | 1543 (8.9) | 368 (5.5) | 327 (5.0) |
| Introduction of Other Gas into Respiratory Tract, Via Natural or Artificial Opening (3E0F7SF) | 1534 (8.8) | 500 (7.4) | 542 (8.3) |
| Introduction of Other Therapeutic Substance into Subcutaneous Tissue, Percutaneous Approach (3E013GC) | 1411 (8.1) | 572 (8.5) | 540 (8.3) |
| Introduction of Analgesics, Hypnotics, Sedatives into Peripheral Vein, Percutaneous Approach (3E033NZ) | 1294 (7.4) | 502 (7.5) | 499 (7.7) |
| Introduction of Other Therapeutic Introduction of Other Therapeutic Substance into Peripheral Vein, Percutaneous Approach (3E033GC) | 993 (5.7) | 306 (4.6) | 347 (5.3) |
| Introduction of Anti-inflammatory into Peripheral Vein, Percutaneous Approach (3E0333Z) | 830 (4.8) | 590 (8.8) | 618 (9.5) |
| Other procedures | 6056 (34.7) | 2620 (39.0) | 2420 (37.1) |

Abbreviations: ICD10 (the 10th revision of the International Statistical Classification of Diseases and Related Health Problems); *Procedures were registered with the ICD-codes
