# Supplementary material for: Healthcare resource use and associated costs in a cohort of hospitalized COVID-19 patients in Spain: A retrospective analysis from the first to the third pandemic wave. EPICOV study
Source: PLoS One. 2023 Jan 25;18(1):e0280940. doi: 10.1371/journal.pone.0280940 (PMC9876243; doi:10.1371/journal.pone.0280940)
Supplement: S5 Table — Recorded only during the hospital ward stay. (DOC) [file pone.0280940.s006.doc]

**S5 Table**. Pharmacological treatments in the different outbreak waves. Recorded only during hospital ward stays.

| **Treatments (ATC4*)** | **Patients, N (%)** | | |
| --- | --- | --- | --- |
|  | **1^st^ Wave**  N = 2066 | **2^nd^ Wave**  N = 661 | **3^rd^ Wave**  N = 667 |
| Solutions affecting the electrolyte balance (B05BB) | 2057 (99.7) | 614 (93.9) | 618 (93.2) |
| Anilides (N02BE) | 2049 (99.3) | 550 (84.1) | 582 (87.8) |
| Solutions for parenteral nutrition (B05BA) | 1963 (95.1) | 167 (25.5) | 141 (21.3) |
| Other antiseptics and disinfectants (D08AX) | 1874 (90.8) | 496 (75.8) | 483 (72.7) |
| Medical gases (V03AN) | 1845 (89.4) | 383 (58.6) | 380 (57.3) |
| Biguanides and amidines (D08AC) | 1831 (88.7) | 217 (33.2) | 212 (32.0) |
| Aminoquinolines (P01BA) | 1814 (87.9) | 17 (2.6) | 1 (0.2) |
| Heparin group (B01AB) | 1707 (82.7) | 606 (92.7) | 612 (92.3) |
| Proton pump inhibitors (A02BC) | 1599 (77.5) | 526 (80.4) | 552 (83.3) |
| Third generation cephalosporins (J01DD) | 1260 (61.0) | 445 (68.0) | 488 (73.6) |
| Macrolides (J01FA) | 1203 (58.3) | 105 (16.1) | 25 (3.8) |
| Antivirals for treatment of HIV infections, combinations (J05AR) | 1070 (51.8) | 0 | 0 |
| Glucocorticoids (H02AB) | 886 (42.9) | 588 (89.9) | 606 (91.4) |

Abbreviations: ATC4 (Anatomical Therapeutic Chemical Classification-Level 4); HIV (human immunodeficiency virus); *Treatments were grouped at the ATC 4^th^ level (chemical subgroup)
