# Supplementary material for: Healthcare resource use and associated costs in a cohort of hospitalized COVID-19 patients in Spain: A retrospective analysis from the first to the third pandemic wave. EPICOV study
Source: PLoS One. 2023 Jan 25;18(1):e0280940. doi: 10.1371/journal.pone.0280940 (PMC9876243; doi:10.1371/journal.pone.0280940)
Supplement: S6 Table — Patients who were not admitted to the ICU. (DOC) [file pone.0280940.s007.doc]

**S6 Table**. Mean costs per patient associated with COVID-19 hospitalization in the different outbreak waves stratified by vaccination age bands and by type of healthcare costs. Patients who were not admitted to the ICU

| **Age groups** | **Waves** | **N (%)** | **Type of healthcare costs** | | | | | | | | | | |
| --- | --- | --- | --- | --- | --- | --- | --- | --- | --- | --- | --- | --- | --- |
|  |  |  | Hospital procedures | | Emergency procedures | | Laboratory test | | Pharmacological treatment | | Hospital LOS | | TOTAL COSTS |
|  |  |  | Mean costs (€) | Percentage of total costs (%) | Mean costs (€) | Percentage of total costs (%) | Mean costs (€) | Percentage of total costs (%) | Mean costs (€) | Percentage of total costs (%) | Mean costs (€) | Percentage of total costs (%) | Mean costs (€) |
| **< 12 years** | **1^st^ Wave** | 5 (0.2) | 174.11 | 5.9 | 12.56 | 0.4 | 65.13 | 2.2 | 30.18 | 1.0 | 2677.30 | 90.5 | 2959.27 |
|  | **2^nd^ Wave** | 3 (0.5) | 241.36 | 11.5 | 13.59 | 0.6 | 0.00 | 0.0 | 9.75 | 0.5 | 1837.36 | 87.4 | 2102.06 |
|  | **3rd Wave** | 1 (0.2) | 1003.58 | 23.1 | 0 | 0.0 | 0.00 | 0.0 | 196.70 | 4.5 | 3149.76 | 72.4 | 4350.04 |
| **12-19 years** | **1^st^ Wave** | 1 (0.0) | 176.98 | 4.1 | 22.00 | 0.5 | 0.00 | 0.0 | 138.35 | 3.2 | 3937.20 | 92.1 | 4274.53 |
|  | **2^nd^ Wave** | 3 (0.5) | 631.38 | 12.6 | 20.93 | 0.4 | 31.00 | 0.6 | 117.46 | 2.3 | 4199.68 | 84.0 | 5000.45 |
|  | **3rd Wave** | 1 (0.2) | 526.50 | 18.2 | 0 | 0.0 | 0.00 | 0.0 | 8.71 | 0.3 | 2362.32 | 81.5 | 2897.53 |
| **20-29 years** | **1^st^ Wave** | 25 (1.2) | 475.13 | 7.5 | 51.76 | 0.8 | 232.04 | 3.6 | 381.92 | 6.0 | 5228.60 | 82.1 | 6369.45 |
|  | **2^nd^ Wave** | 16 (2.4) | 995.35 | 19.4 | 81.83 | 1.6 | 70.60 | 1.4 | 349.78 | 6.8 | 3641.91 | 70.9 | 5139.48 |
|  | **3rd Wave** | 10 (1.5) | 2071.30 | 35.4 | 83.81 | 1.4 | 83.70 | 1.4 | 145.59 | 2.5 | 3464.74 | 59.2 | 5849.13 |
| **30-39 years** | **1^st^ Wave** | 84 (4.1) | 631.65 | 9.8 | 46.80 | 0.7 | 150.04 | 2.3 | 294.18 | 4.6 | 5315.22 | 82.6 | 6437.89 |
|  | **2^nd^ Wave** | 22 (3.4) | 1492.20 | 20.0 | 55.51 | 0.7 | 101.08 | 1.4 | 640.38 | 8.6 | 5189.95 | 69.4 | 7479.12 |
|  | **3rd Wave** | 33 (5.0) | 1490.26 | 25.4 | 16.48 | 0.3 | 81.73 | 1.4 | 157.16 | 2.7 | 4128.10 | 70.3 | 5873.72 |
| **40-49 years** | **1^st^ Wave** | 186 (9.0) | 893.77 | 11.9 | 56.26 | 0.7 | 137.51 | 1.8 | 500.59 | 6.7 | 5931.20 | 78.9 | 7519.34 |
|  | **2^nd^ Wave** | 74 (11.3) | 1251.49 | 15.7 | 48.31 | 0.6 | 117.05 | 1.5 | 600.62 | 7.5 | 5969.65 | 74.7 | 7987.12 |
|  | **3rd Wave** | 83 (12.5) | 1349.61 | 15.8 | 15.38 | 0.2 | 99.92 | 1.2 | 586.78 | 6.9 | 6479.78 | 76.0 | 8531.46 |
| **50-59 years** | **1^st^ Wave** | 310 (15.0) | 1491.17 | 16.1 | 62.72 | 0.7 | 162.84 | 1.8 | 618.44 | 6.7 | 6937.09 | 74.8 | 9272.27 |
|  | **2^nd^ Wave** | 118 (18.0) | 1262.42 | 14.9 | 51.07 | 0.6 | 84.40 | 1.0 | 764.69 | 9.0 | 6319.54 | 74.5 | 8482.11 |
|  | **3rd Wave** | 111 (16.7) | 984.94 | 12.6 | 16.72 | 0.2 | 103.55 | 1.3 | 512.99 | 6.5 | 6228.58 | 79.4 | 7846.78 |
| **60-69 years** | **1^st^ Wave** | 404 (19.6) | 1441.92 | 15.0 | 52.77 | 0.6 | 169.00 | 1.8 | 546.62 | 5.7 | 7383.23 | 77.0 | 9593.53 |
|  | **2^nd^ Wave** | 138 (21.1) | 1286.73 | 12.8 | 61.09 | 0.6 | 96.89 | 1.0 | 1031.99 | 10.3 | 7583.39 | 75.4 | 10 060.10 |
|  | **3rd Wave** | 139 (21.0) | 1417.40 | 15.4 | 23.79 | 0.3 | 100.83 | 1.1 | 711.88 | 7.7 | 6951.00 | 75.5 | 9204.90 |
| **70-79 years** | **1^st^ Wave** | 469 (22.7) | 1376.46 | 12.8 | 53.16 | 0.5 | 230.69 | 2.1 | 828.77 | 7.7 | 8280.71 | 76.9 | 10 769.79 |
|  | **2^nd^ Wave** | 133 (20.3) | 1838.61 | 16.6 | 50.27 | 0.5 | 107.84 | 1.0 | 881.17 | 7.9 | 8211.87 | 74.0 | 11 089.78 |
|  | **3rd Wave** | 143 (21.6) | 2038.80 | 18.1 | 22.29 | 0.2 | 112.67 | 1.0 | 1036.67 | 9.2 | 8028.58 | 71.4 | 11.239.02 |
| **> 80 years** | **1^st^ Wave** | 582 (28.2) | 1813.05 | 16.2 | 85.25 | 0.8 | 388.45 | 3.5 | 542.29 | 4.8 | 8381.77 | 74.8 | 11 210.81 |
|  | **2^nd^ Wave** | 154 (23.5) | 1674.24 | 12.8 | 56.87 | 0.4 | 130.57 | 1.0 | 1184.13 | 9.0 | 10 047.53 | 76.7 | 13 093.34 |
|  | **3rd Wave** | 146 (22.0) | 1396.96 | 13.4 | 21.67 | 0.2 | 107.73 | 1.0 | 719.13 | 6.9 | 8171.04 | 78.4 | 10 416.53 |

Abbreviations: LOS (length of stay)
