# Supplementary material for: Healthcare resource use and associated costs in a cohort of hospitalized COVID-19 patients in Spain: A retrospective analysis from the first to the third pandemic wave. EPICOV study
Source: PLoS One. 2023 Jan 25;18(1):e0280940. doi: 10.1371/journal.pone.0280940 (PMC9876243; doi:10.1371/journal.pone.0280940)
Supplement: S7 Table — Patients admitted to the ICU. (DOC) [file pone.0280940.s008.doc]

**S7 Table**. Mean costs per patient associated with COVID-19 hospitalization in the different outbreak waves stratified by vaccination age bands and by type of healthcare costs. Patients admitted to the ICU

| **Age groups** | **Waves** | **N (%)** | **Type of healthcare costs** | | | | | | | | | | |
| --- | --- | --- | --- | --- | --- | --- | --- | --- | --- | --- | --- | --- | --- |
|  |  |  | Hospital procedures | | Emergency procedures | | Laboratory test | | Pharmacological treatment | | Hospital LOS | | TOTAL COSTS |
|  |  |  | Mean costs (€) | Percentage of total costs (%) | Mean costs (€) | Percentage of total costs (%) | Mean costs (€) | Percentage of total costs (%) | Mean costs (€) | Percentage of total costs (%) | Mean costs (€) | Percentage of total costs (%) | Mean costs (€) |
| **< 12 years** | **1^st^ Wave** | 2 (0.9) | 500.46 | 3.5 | 31.39 | 0.2 | 93.00 | 0.7 | 97.54 | 0.7 | 13 514.91 | 94.9 | 14 237.30 |
|  | **2^nd^ Wave** | 0 | 0.00 | 0.0 | 0.00 | 0.0 | 0.00 | 0.0 | 0.00 | 0.0 | 0.00 | 0.0 | 0.00 |
|  | **3rd Wave** | 0 | 0.00 | 0.0 | 0.00 | 0.0 | 0.00 | 0.0 | 0.00 | 0.0 | 0.00 | 0.0 | 0.00 |
| **12-19 years** | **1^st^ Wave** | 0 | 0.00 | 0.0 | 0.00 | 0.0 | 0.00 | 0.0 | 0.00 | 0.0 | 0.00 | 0.0 | 0.00 |
|  | **2^nd^ Wave** | 0 | 0.00 | 0.0 | 0.00 | 0.0 | 0.00 | 0.0 | 0.00 | 0.0 | 0.00 | 0.0 | 0.00 |
|  | **3rd Wave** | 0 | 0.00 | 0.0 | 0.00 | 0.0 | 0.00 | 0.0 | 0.00 | 0.0 | 0.00 | 0.0 | 0.00 |
| **20-29 years** | **1^st^ Wave** | 2 (0.9) | 20 919.22 | 57.8 | 0.00 | 0.0 | 139.50 | 0.4 | 1047.04 | 2.9 | 14 076.69 | 38.9 | 36 182.44 |
|  | **2^nd^ Wave** | 0 | 0.00 | 0.0 | 0.00 | 0.0 | 0.00 | 0.0 | 0.00 | 0.0 | 0.00 | 0.0 | 0.00 |
|  | **3rd Wave** | 1 (1.4) | 22.00 | 0.2 | 0.00 | 0.0 | 0.00 | 0.0 | 0.74 | 0.0 | 9758.97 | 99.8 | 9781.71 |
| **30-39 years** | **1^st^ Wave** | 1 (0.5) | 823.21 | 5.4 | 62.78 | 0.4 | 90.93 | 0.6 | 1503.13 | 9.9 | 12 740.68 | 83.7 | 15 220.73 |
|  | **2^nd^ Wave** | 0 | 0.00 | 0.0 | 0.00 | 0.0 | 0.00 | 0.0 | 0.00 | 0.0 | 0.00 | 0.0 | 0.00 |
|  | **3rd Wave** | 1 (1.4) | 23 180.29 | 52.3 | 22.00 | 0.0 | 93.00 | 0.2 | 1828.71 | 4.1 | 19 181.84 | 43.3 | 44 305.85 |
| **40-49 years** | **1^st^ Wave** | 16 (7.5) | 27 271.17 | 41.5 | 43.92 | 0.1 | 661.93 | 1.0 | 5574.63 | 8.5 | 32 143.83 | 48.9 | 65 695.49 |
|  | **2^nd^ Wave** | 7 (8.9) | 14 693.91 | 42.9 | 34.47 | 0.1 | 172.71 | 0.5 | 1932.88 | 5.6 | 17 398.44 | 50.8 | 34 232.41 |
|  | **3rd Wave** | 7 (10.0) | 7073.43 | 25.4 | 3.14 | 0.0 | 132.86 | 0.5 | 1772.66 | 6.4 | 18 916.39 | 67.8 | 27 898.48 |
| **50-59 years** | **1^st^ Wave** | 25 (11.7) | 30 279.67 | 38.4 | 55.67 | 0.1 | 1900.89 | 2.4 | 8596.60 | 10.9 | 38 105.07 | 48.3 | 78 937.89 |
|  | **2^nd^ Wave** | 10 (12.7) | 18 992.13 | 34.8 | 19.48 | 0.0 | 139.50 | 0.3 | 5105.62 | 9.4 | 30 260.97 | 55.5 | 54 517.70 |
|  | **3rd Wave** | 15 (21.4) | 7609.00 | 26.4 | 10.05 | 0.0 | 142.60 | 0.5 | 1968.96 | 6.8 | 19 095.73 | 66.2 | 28 826.34 |
| **60-69 years** | **1^st^ Wave** | 81 (38.0) | 31 125.06 | 36.2 | 58.92 | 0.1 | 2112.40 | 2.5 | 9141.97 | 10.6 | 43 626.23 | 50.7 | 86 064.57 |
|  | **2^nd^ Wave** | 20 (25.3) | 29 397.25 | 29.1 | 27.47 | 0.0 | 292.52 | 0.3 | 14 322.12 | 14.2 | 57 091.78 | 56.5 | 101 131.10 |
|  | **3rd Wave** | 19 (27.1) | 18 309.33 | 44.7 | 10.08 | 0.0 | 161.53 | 0.4 | 2758.73 | 6.7 | 19 752.33 | 48.2 | 40 992.00 |
| **70-79 years** | **1^st^ Wave** | 68 (31.9) | 30 561.67 | 35.6 | 48.51 | 0.1 | 2,334.44 | 2.7 | 8981.17 | 10.5 | 43 868.13 | 51.1 | 85 793.91 |
|  | **2^nd^ Wave** | 33 (41.8) | 35 218.66 | 35.2 | 36.50 | 0.0 | 235.62 | 0.2 | 12 056.31 | 12.0 | 52 566.25 | 52.5 | 100 113.40 |
|  | **3rd Wave** | 19 (27.1) | 15 577.36 | 37.2 | 22.62 | 0.1 | 174.37 | 0.4 | 3057.40 | 7.3 | 23 041.33 | 55.0 | 41 873.09 |
| **> 80 years** | **1^st^ Wave** | 18 (8.5) | 11 829.23 | 32.9 | 52.75 | 0.1 | 1266.61 | 3.5 | 3328.08 | 9.3 | 19 452.19 | 54.1 | 35 928.85 |
|  | **2^nd^ Wave** | 9 (11.4) | 11 602.22 | 33.2 | 13.95 | 0.0 | 286.30 | 0.8 | 2515.76 | 7.2 | 20 481.44 | 58.7 | 34 899.66 |
|  | **3rd Wave** | 8 (11.4) | 20 026.00 | 48.5 | 5.10 | 0.0 | 151.13 | 0.4 | 1497.00 | 3.6 | 19 625.37 | 47.5 | 41 304.60 |

Abbreviations: LOS (length of stay)
