# Supplementary material for: Healthcare resource use and associated costs in a cohort of hospitalized COVID-19 patients in Spain: A retrospective analysis from the first to the third pandemic wave. EPICOV study
Source: PLoS One. 2023 Jan 25;18(1):e0280940. doi: 10.1371/journal.pone.0280940 (PMC9876243; doi:10.1371/journal.pone.0280940)
Supplement: S8 Table — (DOC) [file pone.0280940.s009.doc]

**S8 Table**. Results of the sensitivity analysis: mean costs per patient associated with COVID-19 hospitalization in the base case and in the alternative scenarios (minimum costs and maximum costs) in the different outbreak waves.

| **Population** | **Waves** | **Patients not admitted to the ICU** | | | | | | | **Patients admitted to the ICU** | | | | | | |
| --- | --- | --- | --- | --- | --- | --- | --- | --- | --- | --- | --- | --- | --- | --- | --- |
|  |  | **Base case** | Scenario minimum costs | | Scenario maximum costs | | | | | **Base case** | Scenario minimum costs | | Scenario maximum costs | | |
|  |  | Mean costs (€) | Mean costs (€) | Difference from Base case (%) | Mean costs (€) | Difference from Base case (%) | | | | Mean costs (€) | Mean costs (€) | Difference from Base case | Mean costs (€) | | Difference from Base case |
| **< 12 years** | **1^st^ Wave** | 2959.27 | 2062.54 | -30.30 | 3672.92 | | | +24.12 | | 14 237.30 € | 11 363.19 | -20.19 | 17 815.35 | +25.13 | |
|  | **2^nd^ Wave** | 2102.06 | 1498.91 | -28.69 | 2565.36 | | | +22.04 | | - | - | - | - | - | |
|  | **3^rd^ Wave** | 4350.04 | 3316.08 | -23.77 | 5144.28 | | | +18.26 | | - | - | - | - | - | |
| **12-19 years** | **1^st^ Wave** | 3672.92 | 2982.08 | -30.24 | 5267.33 | | | +23.23 | | - | - | - | - | - | |
|  | **2^nd^ Wave** | 2565.36 | 3621.83 | -27.57 | 6059.43 | | | +21.18 | | - | - | - | - | - | |
|  | **3^rd^ Wave** | 5144.28 | 2122.06 | -26.76 | 3493.21 | | | +20.56 | | - | - | - | - | - | |
|  | **1^st^ Wave** | 6369.45 | 4571.76 | -28.22 | 7860.86 | | | +23.41 | | 36 182.44 | 32 555.78 | -10.02 | 39 840.27 | +10.11 | |
| **20-29 years** | **2^nd^ Wave** | 5139.48 | 3938.00 | -23.38 | 6070.49 | | | +18.11 | | - | - | - | - | - | |
|  | **3^rd^ Wave** | 5849.13 | 4711.77 | -19.44 | 6722.79 | | | +14.94 | | 9781.71 | 6862.24 | -29.85 | 12 273.46 | +25.47 | |
|  | **1^st^ Wave** | 6437.89 | 4647.27 | -27.81 | 7875.74 | | | +22.33 | | 15 220.73 | 11 815.57 | -22.37 | 18 707.18 | +22.91 | |
| **30-39 years** | **2^nd^ Wave** | 7479.12 | 5772.25 | -22.82 | 8795.54 | | | +17.60 | | - | - | - | - | - | |
|  | **3^rd^ Wave** | 5873.72 | 4518.61 | -23.07 | 6914.66 | | | +17.72 | | 44 305.85 | 39 713.51 | -10.37 | 49 328.33 | +11.34 | |
| **40-49 years** | **1^st^ Wave** | 7519.34 | 5527.25 | -26.49 | 9108.43 | | | +21.13 | | 65 695.49 | 58 719.00 | -10.62 | 74 892.12 | +14.00 | |
|  | **2^nd^ Wave** | 7987.12 | 6026.50 | -24.55 | 9493.37 | | | +18.86 | | 34 232.41 | 30 550.15 | -10.76 | 38 840.54 | +13.46 | |
|  | **3^rd^ Wave** | 8531.46 | 6401.13 | -24.97 | 10 172.63 | | | +19.24 | | 27 898.48 | 23 149.80 | -17.02 | 32 827.51 | +17.67 | |
| **50-59 years** | **1^st^ Wave** | 9272.27 | 6931.23 | -25.25 | 11 159.37 | | +20.35 | | | 78 937.89 | 69 893.38 | -11.46 | 91 089.97 | +15.39 | |
|  | **2^nd^ Wave** | 8482.11 | 6406.36 | -24.47 | 10 078.58 | | +18.82 | | | 54 517.70 | 47 566.76 | -12.75 | 62 473.09 | +14.59 | |
|  | **3^rd^ Wave** | 7846.78 | 5794.98 | -26.15 | 9433.31 | | +20.22 | | | 28 826.34 | 24 375.90 | -15.44 | 33 839.49 | +17.39 | |
| **60-69 years** | **1^st^ Wave** | 9593.53 | 7100.23 | -25.99 | 11 606.52 | | +20.98 | | | 86 064.57 | 75 848.92 | -11.87 | 99 963.23 | +16.15 | |
|  | **2^nd^ Wave** | 10 060.10 | 7568.08 | -24.77 | 11 978.31 | | +19.07 | | | 101 131.10 | 89 035.28 | -11.96 | 116 254.40 | | +14.95 |
|  | **3^rd^ Wave** | 9204.90 | 6917.61 | -24.85 | 10 969.44 | | +19.17 | | | 40 992.00 | 36 735.67 | -10.38 | 46 215.32 | | +12.74 |
| **70-79 years** | **1^st^ Wave** | 10 769.79 | 7951.84 | -26.17 | 13 075.00 | | +21.40 | | | 85 793.91 | 75 483.07 | -12.02 | 100 002.30 | | +16.56 |
|  | **2^nd^ Wave** | 11 089.78 | 8390.46 | -24.34 | 13 167.78 | | +18.74 | | | 100 113.40 | 88 828.96 | -11.27 | 114 025.10 | | +13.90 |
|  | **3^rd^ Wave** | 11 239.02 | 8599.66 | -23.48 | 13 270.31 | | +18.07 | | | 41 873.09 | 36 623.92 | -12.54 | 47 942.93 | | +14.50 |
| **> 80 years** | **1^st^ Wave** | 11 210.81 | 8270.51 | -26.23 | 13 732.19 | | +22.49 | | | 35 928.85 | 30 632.75 | -14.74 | 42 396.08 | | +18.00 |
|  | **2^nd^ Wave** | 13 093.34 | 9788.54 | -25.24 | 15 641.45 | | +19.46 | | | 34 899.66 | 29 617.52 | -15.14 | 40 246.55 | | +15.32 |
|  | **3^rd^ Wave** | 10 416.53 | 7730.70 | -25.78 | 12 485.11 | | +19.86 | | | 41 304.60 | 37 063.78 | -10.27 | 46 493.06 | | +12.56 |

Abbreviations: ICU (intensive care unit)
