# Supplementary material for: Healthcare resource use and associated costs in a cohort of hospitalized COVID-19 patients in Spain: A retrospective analysis from the first to the third pandemic wave. EPICOV study
Source: PLoS One. 2023 Jan 25;18(1):e0280940. doi: 10.1371/journal.pone.0280940 (PMC9876243; doi:10.1371/journal.pone.0280940)
Supplement: S1 Text — (DOC) [file pone.0280940.s010.doc]

**S1 Text.** Detailed results of the sensitivity analysis

Results of the sensitivity analysis conducted in the population of patients not admitted to the ICU were consistent across outbreak waves and age groups. The analysis of the scenario with the minimum individual costs of laboratory tests and hospital stay resulted in lower mean costs across all the subgroups evaluated. In this scenario, mean costs declined by 19.4% to 30.3% compared to the base case. Meanwhile, the analysis of the scenario with the maximum costs generated higher mean costs that varied among subgroups from an increase of between 14.9% and 24.1% compared to the base case.

Similarly, results for patients admitted to the ICU were also consistent for different outbreak waves and age groups. Thus, when the minimum individual costs were applied, resulting costs declined accordingly by 10.0% to 29.9% compared to the base case. Conversely, with the maximum costs, mean costs increased between 10.1% and 25.5% (**S8 Table**).
