# Supplementary material for: Healthcare resource use and associated costs in a cohort of hospitalized COVID-19 patients in Spain: A retrospective analysis from the first to the third pandemic wave. EPICOV study
Source: PLoS One. 2023 Jan 25;18(1):e0280940. doi: 10.1371/journal.pone.0280940 (PMC9876243; doi:10.1371/journal.pone.0280940)
Supplement: Attachment — Submitted filename: Response to reviewers_V.01_SAN.docx [file pone.0280940.s011.docx]

**MS. Ref. No.: PONE-D-22-09612**

**Title:**

**Healthcare resource use and associated costs of hospitalized COVID-19 patients in Spain: a retrospective analysis from the first to the third pandemic wave. EPICOV study.**

Reviewers’ comments:

**Reviewer #1**

Thank you for the opportunity to review this manuscript on healthcare utilization and cost of COVID-19. While the information on cost of COVID-19 care is important, I believe it is important that it includes adjusted measures of association, in order to answer the questions asked in the paper.

Response to reviewer #1:

We appreciate very much the comments received towards our work. Find below the detailed responses to each of the proposed questions.

1. Page 5, line 97: Please give some insight into how representative these hospitals are of the whole Spain population. What % of total hospitals do they amount to? Are patient or hospital characteristics different? For example, are private hospitals characterized by better standards of care, compared to public ones? It is important in understanding who is represented in this study.

During the first three waves of the pandemic, between 86,000 and 103,000 patients were admitted to hospitals in Spain in each wave. Our study includes data from 3,756 attended in 17 hospitals in four different regions (it is described in lines 96-99). Despite these 17 hospitals belonging to a consortium of private hospitals, due to the dramatic pandemic situation and according to a statal intervention of the private healthcare sector^[1]^, these hospitals stopped their scheduled private care activity to attend to emergency cases referred from public centers that could no longer admit patients. Consequently, the population treated in these centers had the same characteristics as those being treated in the public health system and are therefore representative of Spanish COVID-19 patients.

To clarify this point, we have included in Material and Methods section (lines 99-101) the following text:

*“Due to the pandemic situation, these hospitals stopped their scheduled private care activity to attend to emergency cases referred from public centers that could no longer admit patients.”*

This possible limitation regarding the representativity of the study population is included in the limitation section (lines 486-494): *“our results are reasonably representative of the Spanish healthcare system at the population and cost-analysis level….our database included data from COVID-19 patients in 17 hospital catchment areas across four regions in Spain (Autonomous Community of Madrid, Castilla and Leon, Catalonia, and Galicia), covering an extensive and diverse demographic population. In addition to the extensive geographic area, HM Hospitals put their facilities at the public administration's disposal and absorbed COVID-19 patients from public hospitals during the outbreaks, treating more than 40,000 COVID-19 patients during 2020”.*

Also, we have added (lines 494-496) the following text in this sense:

*“We believe that the main differences between the public and private hospitals prior to the pandemic may be precisely in the length of stay, however, the average length of stay is highly dependent on the pathology [45], which management was equally unknown in both settings. It is important to note that previous studies carried out in Spain in public hospitals during the first wave showed the average length of stay figures similar to those described in our study [46, 47].”*

2. Page 5, line 107: Suggest making this sentence more concise and list the specific date ranges here right away, supported by citations. It does not seem that holiday information (eg Christmas) adds much important information to this.

We welcome suggestions in this regard. In order to make the date ranges for each wave of the pandemic more concise, the paragraph has been modified as follow, and a reference to official sources from the RENAVE epidemiological surveillance system has been incorporated for clarification.

*“The data were grouped according to the RENAVE epidemiological surveillance system into three periods corresponding to the different waves as follows: first wave, from 31st January 2020 to 21st June 2020 [22, 23]; second wave, from 22nd June 2020 to 6th December 2020 [9, 23]; and third wave, from 7th December 2020 to 13th February 2021 [10, 23], and as far as data in our database were available.”* (Lines 106-111)

3. Page 6, line 114: Please provide more information on how the anonymized data were collected. What was the response rate? Are all patients with COVID-19 in these hospitals included?

The database includes data from the Electronic Health Record (EHR) system of the HM Hospitales on patients admitted to the hospitals’ Group. Dataset “covid data save lives” contains the anonymized records of 4,475 patients, admitted with a diagnosis of COVID POSITIVE or COVID PENDING, from the beginning of the epidemic to 13th February 2021. All datasets are linked by a unique admission identifier as a de-anonymization key, created for this purpose, and dissociated from each admission identifier. To provide further information we have included in Material and Methods section the following text on page 6 (Lines 113-121):

*“During the study period (31st January 2020 to 13th February 2021), anonymized data from 4475 COVID-19 patients were recorded in the “COVID Data Saves Lives” dataset [20]. All patients were admitted through the ED. Dataset was extracted from the Electronic Health Record (EHR) system of the HM Hospitals. The information was organized in tables or datasets including information about the COVID-19 treatment process, complete information on admission and diagnoses treatments, ICU admissions, diagnostic imaging tests, laboratory results, drug administration, and cause of discharge/death. All datasets are linked by a unique admission identifier as a de-anonymization key, created for this purpose, and dissociated from each admission identifier.”*

A response rate is not available since all patients included in the datasets were included in the analysis as they met the study criteria.

4. Page 7, line 134: Were patients exclusively admitted through the ED? If so please specify in the text.

Indeed, all patients with suspected or confirmed COVID-19 were admitted through the ED, so we have specified this as appropriate in the methodology section.

*“All patients were admitted through the emergency department (ED).”* (Line 115)

5. Page 7: Was information available on underlying medical conditions?

Most patients included in the dataset were referred from the public health system, therefore previous medical record history is not available. In some cases, when information is known, concomitant diseases are included in the dataset as secondary diagnoses.

6. Page 8: Were any statistical models used? It seems that GLM models with Gamma distribution accounting for specific patient and/or hospital characteristics are needed to compare the costs by these characteristics. All models should be adjusted for age, sex, race/ethnicity, underlying medical conditions (if available) With unadjusted measures of association, it is hard to say whether some of these subpopulations or waves are actually different or whether the difference is driven by other characteristics. For example, the statement in the Conclusions section states that age and ICU admission were the most important drivers of the cost; but it is not possible to assess that without adjusting for all these factors in one model.

We appreciate this suggestion as it enriches our results and conclusions. We have performed additional analysis including these GLM models. Since the distributions of the total cost per patient differ (histogram), a single modelling run may produce inconsistent results, therefore two modelling runs have been performed one for ICU patients and another one for non-ICU patients.

Description of this additional analysis has been included in the Material and Methods section on page 10 (lines 214-223):

*“To identify cost drivers of the covariates in the study population a Generalized Linear Model (GLM) with gamma distribution and log link with stepwise algorithm was used. Outliers in the residuals of the selected model were removed using the interquartile range. In this process, total cost was selected as the dependent variable, and gender, age, wave, and length of stay were independent variables. The estimated coefficients were converted into cost ratios that can be interpreted as a ratio of adjusted costs between the category of interest versus the reference category for binary predictors or as the percentage increase in average cost per unit increase in a continuous covariate. A p-value of less than 0.05 was considered statistically significant. Two modelling runs were performed, one for ICU patients and another one for non-ICU patients.”*

the results of the GLM model for each subgroup (ICU, non-ICU) has been included in Results section (Lines 329-331, non-ICU patients; and 373-377, ICU patients):

*“As a result of the GLM model for non-ICU patients, it was found that age and length of stay significantly increased the total cost per patient by 0.06% and 11,57% per unit respectively.”*

*“GLM model showed that the third wave vs first wave, as well as sex (female vs male) had a significant impact on the total cost per patient decreasing it with respect to the baseline category by 19.58% and 12.97% respectively. On the other hand, in this group, length of stay significantly increased the total cost per patient by 3.48% per unit.”*

We have included this table in response to comments that could clarify the data obtained in the regression.

| Variable | Coef. (exponentiated form) | p-value |
| --- | --- | --- |
| Non ICU-patients | | |
| Age | 1.000593 | 0.025 |
| Length of stay | 1.115735 | 0.000 |
| ICU patients | | |
| Third wave vs fisrt wave | 0.8042193 | 0.007 |
| Sex (Female vs male) | 0.8703277 | 0.043 |
| Length of stay | 1.034786 | 0.000 |

Lastly, we have added the following text in the Discussion section (Lines 458-460):

“*The results of the regression model confirm that length of stay is one of the main factors influencing cost in both ICU and non-ICU populations. In the non-ICU population, age was also an important driver.”*

7. Page 8: Means in this skewed distribution are very much affected by the tail length and outliers; suggest using median costs, in addition to mean costs.

Many thanks to the reviewer for his comment. We have included the medians in table 2 of the manuscript, as attached so that you can observe how means are affected by the tail length and outliers. As shown below, the value of the mean cost is accompanied by the value of the median in brackets (page 16). Also, we have included in Material and Methods the following:

*“For the main cost results, in order to show how the costs are distributed in our population, we also estimate the median cost.”* (lines 192-194).

Table 2 Mean and median costs per patient associated with COVID-19 hospitalization in the different outbreak waves stratified by thy type of healthcare costs.

| **Population** | **Waves** | **N (%)** | **Type of healthcare costs** | | | | | | | | | | |
| --- | --- | --- | --- | --- | --- | --- | --- | --- | --- | --- | --- | --- | --- |
|  |  |  | Hospital procedures | | Emergency procedures | | Laboratory test | | Pharmacological treatment | | Hospital LOS | | **TOTAL COSTS PER PATIENT** |
|  |  |  | Mean (median) costs (€) | Percentage of total costs (%) | Mean (median) costs (€) | Percentage of total costs (%) | Mean (median) costs (€) | Percentage of total costs (%) | Mean (median) costs (€) | Percentage of total costs (%) | Mean (median) costs (€) | Percentage of total costs (%) | **Mean (median) costs (€)** |
| **Patients not admitted to the ICU** | **1^st^ Wave** | 2066 (90.7%) | 1441 (734) | 14.6 | 63 (22) | 0.6 | 241 (93) | 2.4 | 602 (497) | 6.1 | 7 547 (6 300) | 76.3 | **9895** (7 715) |
|  | **2^nd^ Wave** | 661 (89.3%) | 1472 (1014) | 14.4 | 54 (22) | 0.5 | 106 (93) | 1.0 | 902 (224) | 8.9 | 7 661 (6 300) | 75.1 | **10 196** (8 529) |
|  | **3^rd^ Wave** | 667 (90.5%) | 1477 (1046) | 15.8 | 21 (0) | 0.2 | 104 (93) | 1.1 | 696 (1634) | 7.4 | 7 066 (6 300) | 75.5 | **9364** (7 920) |
| **Patients admitted to the ICU** | **1^st^ Wave** | 213 (9.3%) | 28 400 (37 033) | 36.5 | 53 (22) | 0.1 | 1931 (302.6) | 2.5 | 8 070 (4 370) | 10.4 | 39 445 (27 366) | 50.6 | **77 899** (67 343) |
|  | **2^nd^ Wave** | 79 (10.7%) | 27 181 (22 848) | 33.4 | 29 (22) | 0.0 | 238 (186) | 0.3 | 9 766 (4 834) | 12.0 | 44 117 (31 277) | 54.2 | **81 332** (63 170) |
|  | **3^rd^ Wave** | 70 (9.5%) | 14 155 (5 215) | 38.3 | 12 (0) | 0.0 | 154 (139) | 0.4 | 2 375 (1 803) | 6.4 | 20 255 (18 085) | 54.8 | **36 952** (27 930) |

Abbreviations: ICU (intensive care unit); LOS (length of stay)

8. Page 18: The “increase” of LOS and costs with age represents unadjusted means (is that correct?). Suggest using GLM models with age group as the covariate of interest (adjusted for other important controls) to estimate whether this difference was indeed meaningful and significant.

The response to this comment is set out in comment number 6.

9. Methods: Suggest plotting the cost distribution for full sample and/or certain subsets – so that we can understand the skewness and min/max costs.

The response to this comment is set out in comment number 6.

10. Methods: Please consider a supplemental analysis that would censor the costs above 99th percentile. It is possible that means may not be so different after removing those super-utilizers with extremely high costs.

First, thank you very much for your comment. In order to understand the impact of removing those patients with extremely high costs, we have plotted in the following table, by wave and ICU need, the means and medians obtained if we censored the p99 costs, and without censoring them.

| **Wave** | **ICU** | **P99** | **Without super utilizers** | | **With super utilizers** | |
| --- | --- | --- | --- | --- | --- | --- |
|  |  |  | **Mean** | **Median** | **Mean** | **Median** |
| First | No | 39,068 | 9,512 | 7,697 | 9,895 | 7,715 |
|  | Yes | 260,407 | 74,768 | 66,502 | 77,899 | 67,343 |
| Second | No | 38,255 | 9,831 | 8,487 | 10,196 | 8,529 |
|  | Yes | 291,038 | 78,644 | 62,067 | 81,332 | 63,170 |
| Third | No | 37,309 | 9,032 | 7,877 | 9,365 | 7,920 |
|  | Yes | 106,775 | 35,940 | 27,883 | 36,952 | 27,930 |

As can be seen, the data indicate that these super-utilizers with extremely high costs do not interfere to a large extent with the mean/median cost. Taking into account the volume of results already provided in the manuscript, we consider that these data, despite their statistical interest, do not provide further insight into the manuscript and would make it difficult to understand.

11. Methods and Results: While mean costs are interesting, it would also be interesting to understand the specific contributions of all patient characteristics to cost in terms of percent. For example, as age increases, what is the increase in costs in percent? This gives us some understanding of the magnitude of this difference (2% or 50%), rather than just dollar terms.

The response to this comment is set out in comment number 6.

12. Figures: Please add footnotes describing what is shown in each figure (whether these are adjusted or unadjusted cost means). Each figure must stand alone and be interpretable without looking at the Methods.

Thank you for your comment. We have considered appropriate to include this information in the footnotes, as can be seen on page 18 for figures 3 and 4.

*“Costs are estimated as unadjusted means.”*

**Reviewer #2**

Response to reviewer #2:

We appreciate very much the comments received towards our work. Find below the detailed responses to each of the proposed questions.

Major issues:

1. There is already a paper which present costs related to cost of COVID cases. The authors cited it <https://healtheconomicsreview.biomedcentral.com/articles/10.1186/s13561-021-00340-0>

Then what is the added value of the current paper?

First of all, we thank you for your comments on this manuscript which we consider to be of great interest. However, while this paper discusses the economic impact of hospitalization in a single tertiary hospital during the first wave, with an analysed population size of 1602 patients, our analysis includes data from different hospitals, in different geographical areas, with a larger sample, extending the analysis to the first three waves, which provides a more complete picture of the first year of the pandemic.

2. In my view it is not ok to mix cost and mortality. Your paper deals mainly with cost and there are a lot of information that you can present about costs. I recommend to make a different paper in mortality/survival, etc.

We appreciate the suggestion. We agree that the information on mortality or survival does not provide relevant information for the cost analysis. However, data on the number of patients who died may allow describing the flow of our population from admission to discharge, so we have included this data in fig 2.

According to your comment, we have removed the mortality section from the manuscript.

3. The paper has rather a descriptive manner, rather a case-study. If the sample is random, I suggest to make an inference of COVID cost’s

The study sample was not randomly selected, therefore we would not be able to do inferential statistics. The origin of the data has been detailed in this new version of the manuscript.in Material and Methods section the following text on page 6 (Lines 113-121) :

*“During the study period (31st January 2020 to 13th February 2021), anonymized data from 4475 COVID-19 patients were recorded in the “COVID Data Saves Lives” dataset [20]. All patients were admitted through the ED. Dataset was extracted from the Electronic Health Record (EHR) system of the HM Hospitals. The information was organized in tables or datasets including information about the COVID-19 treatment process, complete information on admission and diagnoses treatments, ICU admissions, diagnostic imaging tests, laboratory results, drug administration, and cause of discharge/death. All datasets are linked by a unique admission identifier as a de-anonymization key, created for this purpose, and dissociated from each admission identifier.”*

4. Related to (1), (3). Probably is more important to see what combination of factors/covariates drive to higher costs? This can be an added value of the paper. In this sense, I suggest to use for example regression modelling.

We appreciate this suggestion as it enriches our results and conclusions. We have performed additional analysis including these GLM models. Since the distributions of the total cost per patient differ (histogram), a single modelling run may produce inconsistent results, therefore two modelling runs have been performed one for ICU patients and another one for non-ICU patients.

Description of this additional analysis has been included in the Material and Methods section on page 10 (lines 214-223):

*“To identify cost drivers of the covariates in the study population a Generalized Linear Model (GLM) with gamma distribution and log link with stepwise algorithm was used. Outliers in the residuals of the selected model were removed using the interquartile range. In this process, total cost was selected as the dependent variable, and gender, age, wave, and length of stay were independent variables. The estimated coefficients were converted into cost ratios that can be interpreted as a ratio of adjusted costs between the category of interest versus the reference category for binary predictors or as the percentage increase in average cost per unit increase in a continuous covariate. A p-value of less than 0.05 was considered statistically significant. Two modelling runs were performed, one for ICU patients and another one for non-ICU patients.”*

the results of the GLM model for each subgroup (ICU, non-ICU) has been included in Results section (Lines 329-331, non-ICU patients; and 373-377, ICU patients):

*“As a result of the GLM model for non-ICU patients, it was found that age and length of stay significantly increased the total cost per patient by 0.06% and 11,57% per unit respectively.”*

*“For ICU patients, the third wave vs first wave as well as sex (female vs male) had a significant impact on the total cost per patient decreasing it with respect to the baseline category by 19.58% and 12.97% respectively. On the other hand, in this group, length of stay significantly increased the total cost per patient by 3.48% per unit.”*

We have included this table in response to comments that could clarify the data obtained in the regression.

| Variable | Coef. (exponentiated form) | p-value |
| --- | --- | --- |
| Non ICU-patients | | |
| Age | 1.000593 | 0.025 |
| Length of stay | 1.115735 | 0.000 |
| ICU patients | | |
| Third wave vs fisrt wave | 0.8042193 | 0.007 |
| Sex (Female vs male) | 0.8703277 | 0.043 |
| Length of stay | 1.034786 | 0.000 |

Lastly, we have added the following text in the Discussion section (Lines 458-460):

“*The results of the regression model confirm that length of stay is one of the main factors influencing cost in both ICU and non-ICU populations. In the non-ICU population, age was also an important driver.”*

5. You present some results about sensitivity, but in the methods the sensitivity analysis is described too vague. It is DeterministicSA or ProbabilitySA, I guess is DSA.

Indeed, we have carried out a deterministic analysis as we only vary the costs by a percentage. In order to make it clear how the sensitivity analysis has been carried out, we have added in the methodology the following:

*“A deterministic sensitivity analysis was conducted to estimate the impact of selecting particular costs rather than others in the cost analysis results.“* (Lines 225-226)

6. You deal with samples in each wave, why you do not test if the difference is statistically significant? Between waves and or between groups of patients.

Thank you very much for pointing this out. The aim of the project was purely descriptive of resource utilization and associated costs, with no intention of comparison. Regardless of this, the sample size might be insufficient to make comparisons.

7. We know that LOS and age are determinants for higher costs, but it is something else a characteristic of COVID hospitalized cases? If you want to add a value to your study, why not a comparison with severe classic Pneumonia cases? If the cost are not significantly higher, than….

The response to this comment is set out in comment number 4.

Regarding the question about including a comparison with severe pneumonia cases, this could be very timely to address or discuss in future work, however, the management of COVID in the context of the beginning of the pandemic, when the disease was barely known and clinical management was adapted as more information became available, in our view makes it difficult to compare it with a well-known pathology, with well-established standards of care.

8. In the sense of (7) I suggest to search for a combination of factors such as, age-group+ group of comorbidities.

This comment has been addressed in comment number 4.

9. Can we see a distribution of patients by cost? I believe is a long tail distribution, then the mean cost may be not relevant….Eg. you sum 3patients*2000 +1patients *10000 and you have a mean of 4000. The range of cost is not enough relevant.

We appreciate the reviewer's comment, in line with the comments of reviewer 1, we have responded to this comment on points 7 and 10 above.

10. Maybe a synthetic 8 figure/diagram is useful to understand the patient path-way. Eg diagnosed at family medicine- covid ward- ICU, or Non-diagnosed----emergency---ICU.

This is a fantastic idea, and we believe that, in line with comment 2, we can describe the patient flow and include the mortality figures that we think is a relevant information, without going as deep into mortality as we do. We are considering the possibility of publishing another manuscript more focused on comorbidities, mortality and survival. We have included a figure (Fig 2, Page 12, line 249) representing the patient journey and the volume of patients in each state.

*Fig 2. Inpatient pathway of the study cohort.*

11. For the hospital costs we have a DRG cost? Then, the structure of patients per hospital is the same in each wave? I think we have a hospital or regional effect cost when DRG is computed. Please clarify!

Thank you for your comment, in this case, due to the large number of different unit costs collected for the patients, those that were available were used as it was not possible to use for all of them DRGs or regional tariffs. Precisely to analyze the possible effect on the results of the selection of costs, the sensitivity analysis was performed considering the minimum and maximum.

Minor issues

1. Reference [27] has a broken link

Thank you very much for the warning, the link must have been updated. We have modified it so that the reader has access.

2. If you decide to keep mortality in the analysis then, in the methodology some description, formula or something else should appear about mortality. Then if then, maybe you should take into account

Finally, as you suggested, we have removed the section on mortality.

3. I don’t know if it so important to use so many age-groups . The difference are small. Using so many age-groups you reduce sub-sample size. Why not children? (<18)S, Then 18-29, 30-49, 50-69, 70+, or something similar.

The criterion followed for the estimation by age subgroups is based on vaccination schedules (*Both mean costs per patient and cost per patient and day were stratified by the vaccination age bands in Spain: < 12, 12-19, 20-29, 30-39, 40-49, 50-59, 60-69, 70-79, and > 80 years*). Other criteria could have been followed, perhaps arbitrarily, but it seemed to us an appropriate criterion because at the time of the analysis mass vaccination was being initiated in Spain, with uncertainty in some age groups. This uncertainty about the procedure for systematic and temporary vaccination and prevention of the virus is still ongoing. Increasing the age ranges could give us subgroups with larger samples, however, our analysis was not intended to compare between age ranges but to describe how the costs are distributed so that the reader can have more concrete data on each sub-population.

4. Secondary diagnosis at hospital admission, n/N (%) please use other notation, Usually n= the sample size and N is the population size.

We appreciate very much this suggestion to clarify these data. In order to differentiate it from n/N, we have proposed to use d/D where, d is the number of secondary diagnoses identified with that code and D is the number of total secondary diagnoses for our study population (including all codes). Accordingly, we have modified Table 1 (Page 12-13).

5. Secondary diagnosis at hospital admission, n/N (%). I don’t understand the shares. In my view we should see how many COVID patients have an I10 or E11 as secondary diagnostic. Now I think it is a share of how many I10 are in the secondary diagnostics divided by patients*nr_of secondary diagnostics ?!. Please clarify!

Thank you for your comment. Given the number of secondary diagnoses on admission, it was decided to group them by diagnostic groups and therefore the percentage obtained is given by number of secondary diagnoses in each group/total secondary diagnoses. The data shall be clarified as indicated in point 4.

Due to the relevance or prevalence of hypertension and diabetes in the general population, we have considered it necessary to differentiate the number of patients with a secondary diagnosis for these two specific pathologies (n/N), while the rest of the diagnoses have been grouped together (d/D).

If it is considered relevant for the description of the sample, the percentage of patients with secondary diagnosis I10 and E11.9 has been added.

| **Diagnosis** | **First wave** | **Second wave** | **Third wave** |
| --- | --- | --- | --- |
| I10, n/N (%) | 809/2279 (35.5%) | 251/740 (33.9%) | 260/737 (35.3%) |
| E11.9, n/N (%) | 249/2279 (10.9%) | 72/740 (9.7%) | 64/737 (8.7%) |

Accordingly, we have modified Table 1 (Page 12-13).

1. Royal Decree 463/2020 of 14 March declaring a state of alarm for the management of the health crisis situation caused by COVID-19. Available in: <https://www.boe.es/buscar/doc.php?id=BOE-A-2020-3692>
