# Supplementary material for: Healthcare resource use and associated costs in a cohort of hospitalized COVID-19 patients in Spain: A retrospective analysis from the first to the third pandemic wave. EPICOV study
Source: PLoS One. 2023 Jan 25;18(1):e0280940. doi: 10.1371/journal.pone.0280940 (PMC9876243; doi:10.1371/journal.pone.0280940)
Supplement: Attachment — Submitted filename: Response to reviewers_2_V.02.docx [file pone.0280940.s012.docx]

**MS. Ref. No.: PONE-D-22-09612 R1**

**Title:**

**Healthcare resource use and associated costs of hospitalized COVID-19 patients in Spain: a retrospective analysis from the first to the third pandemic wave. EPICOV study.**

Journal Requirements:

Please review your reference list to ensure that it is complete and correct. If you have cited papers that have been retracted, please include the rationale for doing so in the manuscript text, or remove these references and replace them with relevant current references. Any changes to the reference list should be mentioned in the rebuttal letter that accompanies your revised manuscript. If you need to cite a retracted article, indicate the article’s retracted status in the References list and also include a citation and full reference for the retraction notice.

Reviewers' comments:

Comments to the Author

1. If the authors have adequately addressed your comments raised in a previous round of review and you feel that this manuscript is now acceptable for publication, you may indicate that here to bypass the “Comments to the Author” section, enter your conflict of interest statement in the “Confidential to Editor” section, and submit your "Accept" recommendation.

Reviewer #1: (No Response)

Reviewer #2: (No Response)

________________________________________

2. Is the manuscript technically sound, and do the data support the conclusions?

The manuscript must describe a technically sound piece of scientific research with data that supports the conclusions. Experiments must have been conducted rigorously, with appropriate controls, replication, and sample sizes. The conclusions must be drawn appropriately based on the data presented.

Reviewer #1: Partly

Reviewer #2: No

________________________________________

3. Has the statistical analysis been performed appropriately and rigorously?

Reviewer #1: No

Reviewer #2: No

________________________________________

4. Have the authors made all data underlying the findings in their manuscript fully available?

The PLOS Data policy requires authors to make all data underlying the findings described in their manuscript fully available without restriction, with rare exception (please refer to the Data Availability Statement in the manuscript PDF file). The data should be provided as part of the manuscript or its supporting information, or deposited to a public repository. For example, in addition to summary statistics, the data points behind means, medians and variance measures should be available. If there are restrictions on publicly sharing data—e.g. participant privacy or use of data from a third party—those must be specified.

Reviewer #1: No

Reviewer #2: Yes

________________________________________

5. Is the manuscript presented in an intelligible fashion and written in standard English?

PLOS ONE does not copyedit accepted manuscripts, so the language in submitted articles must be clear, correct, and unambiguous. Any typographical or grammatical errors should be corrected at revision, so please note any specific errors here.

Reviewer #1: Yes

Reviewer #2: Yes

________________________________________

6. Review Comments to the Author

**Reviewer #1:**

I appreciate the authors’ responses to my comments. While most of my comments and questions have been resolved, I have two that have not been fully resolved. My focus is especially on the **adjusted measures of association**; although authors have provided them, I feel that the table/figure needs to be included in the manuscript and explained in more detail as well.

Response to reviewer #1:

We appreciate very much the comments received towards our work. Find below the detailed responses to each of the proposed questions.

R1, Comment 1: I feel that my comment about the representativeness was only partially addressed. Could the authors add in the text the total number of hospitals in Spain that were open during the pandemic, as well as the % that the 17 hospitals included in the study represent? I appreciate the added limitation, but quantifying the representativeness would be helpful here.

We welcome suggestions in this regard. Even though the hospitals included are only a small sample of hospitals in the Spanish health system (2.2% of all Spanish hospitals, public and private), we suggest that the patients included in this study could represent the reality of the health system during the first waves of the pandemic. However, the reader could interpret this as inferential statistics, which is not our objective, but rather describe what happens in our study population. For this reason, we wanted to clarify and highlight at some points in the manuscript that our findings refer to the studied sample.

In this regard, we have introduced the following changes in different sections of the manuscript:

- Title of the manuscript to: *“Healthcare resource use and associated costs* ***in a cohort of hospitalized COVID-19 patients*** *in Spain: a retrospective analysis from the first to the third pandemic wave. EPICOV study.”*
- Conclusions section from the Abstract (line 39): *“LOS was longer for patients admitted to the ICU (especially in the first two waves) and for older patients* ***in our study cohort****; these populations incurred the highest hospitalization costs.”*
- Introduction section (lines 81-82): *“In the present study, we aimed to use real-world data to describe the use of healthcare resources and the associated costs* ***in a cohort of hospitalized patients*** *due to SARS-CoV-2 during three different outbreak waves from the perspective of Spain's national health system (NHS).”*
- Discussion section:

Lines 396-396: *“We have described the use of healthcare resources and costs* ***in a cohort of hospitalized COVID-19 patients*** *in Spain”*

Line 432: *“We should also point out that mean costs per patient and per day* ***in our study cohort*** *did not differ substantially between the outbreak waves”*

Lines 449-450: *“In addition, the treatments administered to patients differed drastically between the first wave and the other two. In this respect, during the first wave, most patients* ***in our sample*** *were treated with aminoquinolines and antivirals”*

Lines 458-459: *“Another interesting observation was that mean costs per COVID-19 patient and, especially, the LOS increased with age* ***in our study cohort****.”*

Line 464: *“The results of the regression model confirm that length of stay is one of the main factors influencing cost in both ICU and non-ICU populations* ***of our study sample****.”*

Lines 486-492: *“Our cost analysis has some limitations that should be mentioned. First, the data were extracted from the HM Hospitals database, which agglutinated data from a consortium of private hospitals in Spain.* ***Furthermore, this sample of hospitals represented 2.2% of Spanish hospitals (41)****. In that respect, the study population might not accurately represent the population attended in the Spanish healthcare system. Nonetheless,* ***we consider*** *that our results* ***could be*** *reasonably representative of the Spanish healthcare system at the population and cost-analysis level.”*

A reference has been included to support the included text: Hospital statistics - National tables (2020). [Cited 7 December 2022]. In: Spanish Ministry of Health [Internet]. Available from: https://www.sanidad.gob.es/estadEstudios/estadisticas/docs/TablasSIAE2020/Tablas_Nacionales_2020.pdf

Regarding this point, we would like to point out that a sensitivity analysis was carried out taking into account a possible under- or overestimation of the cost.

Lines 506-508: *“Therefore,* ***we suggest that the costs obtained are an approximation of the actual costs*** *of the Spanish public healthcare system.”*

- Conclusions section:

Lines 530-534: “This study describes the use of healthcare resources and costs **in a cohort of hospitalized COVID-19 patients** over the course of the first year of the pandemic. Accordingly, our main observation is that mean costs per hospitalization COVID-19 patient did not consistently differ across the three main outbreak waves **in our sample**, especially for those patients who were not admitted to the ICU”

Lines 540-541*: “In summary, our cost analysis provides robust data* ***that could be useful*** *to inform decision-makers about how health systems were impacted across the different outbreak waves in the first year of the COVID-19 pandemic.”*

R1, Comment 6: I appreciate that the authors addressed my comment by using adjusted GLM models and providing adjusted measures of association in the text. I have some more comments on the modeling, however.

More comments below:

- Estimates need to include confidence intervals.

Thank the reviewer for this comment. We have added the confidence intervals in the manuscript in section Results, lines 333-334 and lines 378-380.

To facilitate the interpretation of the results obtained, they have been included in the form of percentages, so that the explanation of the coefficients obtained is more intuitive for the reader.

| Variable | | Coef. (exponentiated form) | Std. Error | p-value | IC 95% | |
| --- | --- | --- | --- | --- | --- | --- |
|  | Non ICU-patients | | | | | |
| Age | | 1.000593 | 0.000265 | 0.025 | 1.000074 | 1.001113 |
| Length of stay | | 1.115735 | 0.0012762 | 0.000 | 1.113236 | 1.118239 |
|  | ICU patients | | | | | |
| Third wave vs fisrt wave | | 0.8042193 | 0.0651603 | 0.007 | 0.6861319 | 0.9426303 |
| Second wave vs fisrt wave | | 1.00022 | 0.0769607 | 0.998 | 0.8602026 | 1.163028 |
| Sex (Female vs male) | | 0.8703277 | 0.0595943 | 0.043 | 0.7610236 | 0.995331 |
| Length of stay | | 1.034786 | 0.0021719 | 0.000 | 1.030538 | 1.039051 |

- Why aren’t all estimates shown in the table in the authors’ responses? I see only age and LOS as predictors of cost among non-ICU patients and wave (but only third wave vs first wave), sex, and LOS as a predict among ICU patients. I would suggest that all coefficients are presented.

A stepwise GLM model was used to carry out the estimation; this type of modelling eliminates those factors that are not significant from the estimation. For predictors whose p-value is greater than 0.05, no coefficient is obtained.

In the case of ICU patients, only the third wave vs. the first wave was significant. However as a categorical variable, the coefficient for the second wave vs. the first wave was estimated too, as it should be included in the modelling.

We have added an additional more detailed explanation of Stepwise in the manuscript in section Analysis, lines 215-219:

*“To identify cost drivers of the covariates in the study population a Generalized Linear Model (GLM) with gamma distribution and log link with stepwise algorithm was used.* ***The stepwise regression consists of iteratively adding and removing predictors, in the predictive model, in order to find the subset of variables in the data set resulting in the best performing model, that is a model that lowers prediction error.****”*

- Even more important than coefficients, predicted costs from the GLM models can be used in the place of unadjusted mean costs in the figures. Their 95% Cis can show whether the costs in the three waves were truly different.

We are very grateful to the reviewer for this and the following two comments, we consider that including the GLM model in the publication has helped us to compare costs according to different population characteristics and has reinforced the conclusions that a priori were drawn from a purely descriptive analysis. However, modifying the mean costs obtained by post-estimation of these through the coefficients, we believe that it modifies the descriptive objective of the project and would imply completely restructuring the publication. The aim of this analysis is not to model the results and thus smooth out heterogeneity, but to evaluate and describe the results obtained directly by the patient, so we consider that presenting the median helped to present greater detail in this respect. Even though this estimation would be of real interest, it may require a refocusing of the project. This would not only include additional analyses but replace the analysis that we have carried out as the aim of the study. Moreover, including all this analysis would overextend and complicate the interpretation of the results.

- Are these estimates currently included in the main figures? If estimates are not included in the main results, I would suggest replacing one of the figures with the adjusted predicted costs (and 95% CI) in each wave. That can be easily done by using the “margins” Stata post-estimation command to get predicted costs. This would help us see if the costs were truly different by wave after controlling for other covariates.

- To estimate differences by wave for each characteristic (Figure 3A), authors may want to include both wave categorical variable and its interaction with those characteristics (eg wave (1,2,3), age (<12, 12-19, 20-29, 30-39, 40-49, 50-59, 60-69, 70-79, 80), and wave*age). They can then use the Stata “margins” post-estimation command to predict the cost in each wave and at each level of covariate included in the interaction.

**Reviewer #2:**

I appreciate the work of authors but there are some issue which in my view do not match.

The major thing is that the title, the abstract and the conclusions suggest that achieved results characterize the cost/patient for entire population of COVID-19 patient in Spain. The sample selection and thus the methods and the results do not support this hypothesis. In my view there two options (1) if the authors try to extend the costs results for a typical ICU/non-ICU patient from Spain, then they need a representative sample; (2) if the authors cannot achieve a representative sample, I recommand to underline the fact, that this is work is just a case-study, and everywhere in the paper should be clearly specified that "in our/current sample"/ "in our/current case", the results are. The phrases which induce the extension of results should be avoided in my view. Indeed in the limitations paragraph the authors try to explain differences between the current sample and the population, but the arguments are not solid. My worries are related to the thing that costs may be overestimated in particular hospitals compared with anothers. There is no proof that error are compensating each-other.

Response to reviewer #2:

Many thanks to the reviewer for his comments as we consider that they have improved the manuscript. The changes implemented in response to the concerns raised by the reviewer are included below.

We have introduced the following changes in different sections of the manuscript:

- Title of the manuscript to: *“Healthcare resource use and associated costs* ***in a cohort of hospitalized COVID-19 patients*** *in Spain: a retrospective analysis from the first to the third pandemic wave. EPICOV study.”*
- Conclusions section from the Abstract (line 39): *“LOS was longer for patients admitted to the ICU (especially in the first two waves) and for older patients* ***in our study cohort****; these populations incurred the highest hospitalization costs.”*
- Introduction section (lines 81-82): *“In the present study, we aimed to use real-world data to describe the use of healthcare resources and the associated costs* ***in a cohort of hospitalized patients*** *due to SARS-CoV-2 during three different outbreak waves from the perspective of Spain's national health system (NHS).”*
- Discussion section:

Lines 396-396: *“We have described the use of healthcare resources and costs* ***in a cohort of hospitalized COVID-19 patients*** *in Spain”*

Line 432: *“We should also point out that mean costs per patient and per day* ***in our study cohort*** *did not differ substantially between the outbreak waves”*

Lines 449-450: *“In addition, the treatments administered to patients differed drastically between the first wave and the other two. In this respect, during the first wave, most patients* ***in our sample*** *were treated with aminoquinolines and antivirals”*

Lines 458-459: *“Another interesting observation was that mean costs per COVID-19 patient and, especially, the LOS increased with age* ***in our study cohort****.”*

Lines 464: *“The results of the regression model confirm that length of stay is one of the main factors influencing cost in both ICU and non-ICU populations* ***of our study sample****.”*

Lines 486-492: *“Our cost analysis has some limitations that should be mentioned. First, the data were extracted from the HM Hospitals database, which agglutinated data from a consortium of private hospitals in Spain.* ***Furthermore, this sample of hospitals represented 2.2% of Spanish hospitals****. In that respect, the study population might not accurately represent the population attended in the Spanish healthcare system. Nonetheless,* ***we consider*** *that our results* ***could be*** *reasonably representative of the Spanish healthcare system at the population and cost-analysis level…”*

A reference has been included to support the included text: Hospital statistics - National tables (2020). [Cited 7 December 2022]. In: Spanish Ministry of Health [Internet]. Available from: <https://www.sanidad.gob.es/estadEstudios/estadisticas/docs/TablasSIAE2020/Tablas_Nacionales_2020.pdf>

Regarding this point, we would like to point out that a sensitivity analysis was carried out taking into account a possible under- or overestimation of the cost.

Lines 506-508: *“Therefore,* ***we suggest that the costs obtained are an approximation of the actual costs*** *of the Spanish public healthcare system.”*

- Conclusions section:

Lines 530-534: “This study describes the use of healthcare resources and costs **in a cohort of hospitalized COVID-19 patients** over the course of the first year of the pandemic. Accordingly, our main observation is that mean costs per hospitalization COVID-19 patient did not consistently differ across the three main outbreak waves **in our sample**, especially for those patients who were not admitted to the ICU”

Lines 540-541*: “In summary, our cost analysis provides robust data* ***that could be useful*** *to inform decision-makers about how health systems were impacted across the different outbreak waves in the first year of the COVID-19 pandemic.”*
